# Nematode Homologue of PQBP1, a Mental Retardation Causative Gene, Is Involved in Lipid Metabolism

**DOI:** 10.1371/journal.pone.0004104

**Published:** 2009-01-01

**Authors:** Keiko Takahashi, Sawako Yoshina, Maekawa Masashi, Wakana Ito, Takao Inoue, Hiroki Shiwaku, Hiroyuki Arai, Shohei Mitani, Hitoshi Okazawa

**Affiliations:** 1 Department of Neuropathology, Medical Research Institute, Tokyo Medical and Dental University, Tokyo, Japan; 2 Department of Physiology, Tokyo Women's Medical University School of Medicine, Tokyo, Japan; 3 Department of Health Chemistry, Graduate School of Pharmaceutical Sciences, The University of Tokyo, Tokyo, Japan; Massachusetts Institute of Technology, United States of America

## Abstract

**Background:**

*PQBP1* is a causative gene for X-linked mental retardation (MR) whose patients frequently show lean body. C. elegans has a strictly conserved homologue gene of *PQBP1*, *T21D12.3*.

**Methodology and Principal Findings:**

We generated *Venus*-transgenic and *T21D12.3-*mutant nematodes to analyze developmental expression patterns and *in vivo* functions of the nematode PQBP1 homologue protein (pqbp-1.1). During development, pqbp-1.1 is expressed from cell proliferation stage to larva stage. In larva, intestinal cells show the highest expression of pqbp-1.1, while it decreases in adult worms. The mutants of pqbp-1.1 show a decrease of the lipid content in intestinal cells. Especially, incorporation of fatty acid into triglyceride is impaired. ShRNA-mediated repression of PQBP1 also leads to reduction of lipid content in mammalian primary white adipocytes.

**Conclusion/ Significance:**

These results suggest that pqbp-1.1 is involved in lipid metabolism of intestinal cells. Dysfunction of lipid metabolism might underlie lean body, one of the most frequent symptoms associating with *PQBP1*-linked MR patients.

## Introduction

PQBP1 was identified as a binding protein to the polyglutamine (polyQ) tract of the causative gene products of a major group of neurodegeneration, polyglutamine diseases [1]. PQBP1 includes two protein interaction domains: WW domain (WWD) through which PQBP1 interacts with RNA polymerase II and NpwBP [2], [3] and C-terminal domain (CTD) through which it interacts with a splicing factor, U5-15kD [4], [5]. Subsequently, PQBP1 was identified as a causative gene for a group of mental retardation including Renpenning syndrome, Golabi-Ito-Hall and the Sutherland-Haan syndromes as well as non-symptomatic X-linked mental retardation with no anomaly [6]–[8]. The frequency of these PQBP1-linked disorders is relatively high [9]. Among various associating symptoms, one of the most frequent symptoms in these patients is lean body, which is found in 87% of the patients [10].

In this study, we investigated expression patterns of nematode homologue of PQBP1 (pqbp-1.1) during development, and generated mutants of *T21D12.3*. We found that pqbp-1.1 is expressed in intestinal cells storing lipid in nematodes and that the mutant of *T21D12.3* shows reduction of lipid storage in intestinal cells although intestinal cells did not reduce in their number. Furthermore, we found shRNA-mediated knocking down of PQBP1 leads to reduction of lipid storage in mouse primary white adipocytes. These results suggest that PQBP1 is involved in lipid metabolism beyond species and that dysfunction of lipid metabolism might be a cause of lean body in human PQBP1 mutations.

## Results

### PQBP1 homologue is expressed in intestinal cells

**Figure 1:**
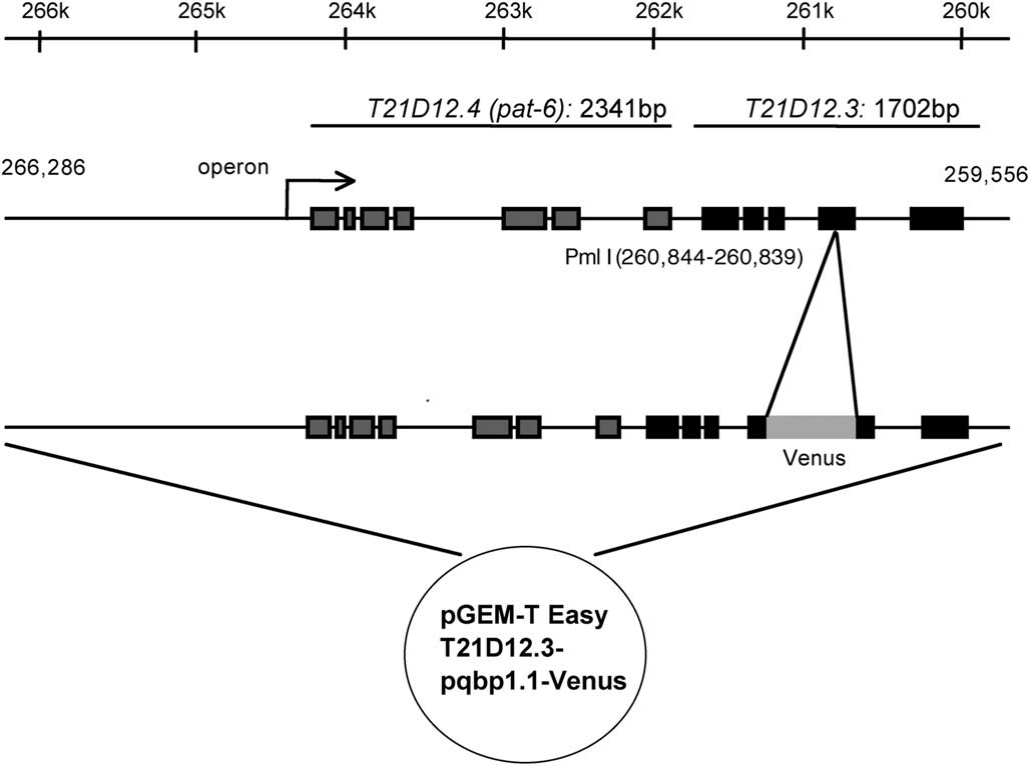
Construction of transgenic C. elegans expressing *Venus* under the control of *T21D12.3* enhancer/promoter. *T21D12.3* and *T21D12.4* (*pat-6*) are transcriptionally regulated as an operon. Therefore, 6.7 kb genome DNA from the transcriptional initiation site to the neighboring gene to *pat-6* was subcloned into pGEMT-T plasmid. By injecting the plasmid into germ line of adult worm, we obtained transgenic nematodes in which the Venus cDNA was subcloned into exon 4 to express a PQBP1-Venus fusion protein under the control of endogenous enhancer/promoter.

C. elegans possesses a homologue gene of PQBP1, *T21D12.3*, which encodes a protein (pqbp-1.1) containing both of the essential protein interacting domains, WWD and CTD [11]. Amino acid identities between human and nematode PQBP1 are 35% in the whole molecule, 52% in WWD and 55% in CTD. To analyze expression patterns of *T21D12.3*, the PQBP1 homologue in C. elegans, we constructed a reporter plasmid expressing a fluorescent fusion protein of pqbp-1.1 and Venus, a variant of yellow fluorescent protein with fast and efficient maturation for visualization [12] under the control of *T21D12.3* enhancer/promoter according to the method described previously [13]. Endogenously, *T21D12.3* and *T21D12.4* (*pat-6*) are regulated as an operon (Fig. 1). Therefore, we constructed a plasmid that contains 6.7 kb genome DNA from the 2.7 kb upstream of the neighboring gene (*pat-6*) to the 3′ end of *T21D12.3* (Fig. 1). Thereafter, Venus cDNA was subcloned into exon 4 of *T21D12.3* to express a nematode PQBP1-Venus fusion protein (pqbp-1.1-Venus).

**Figure 2 pone-0004104-g002:**
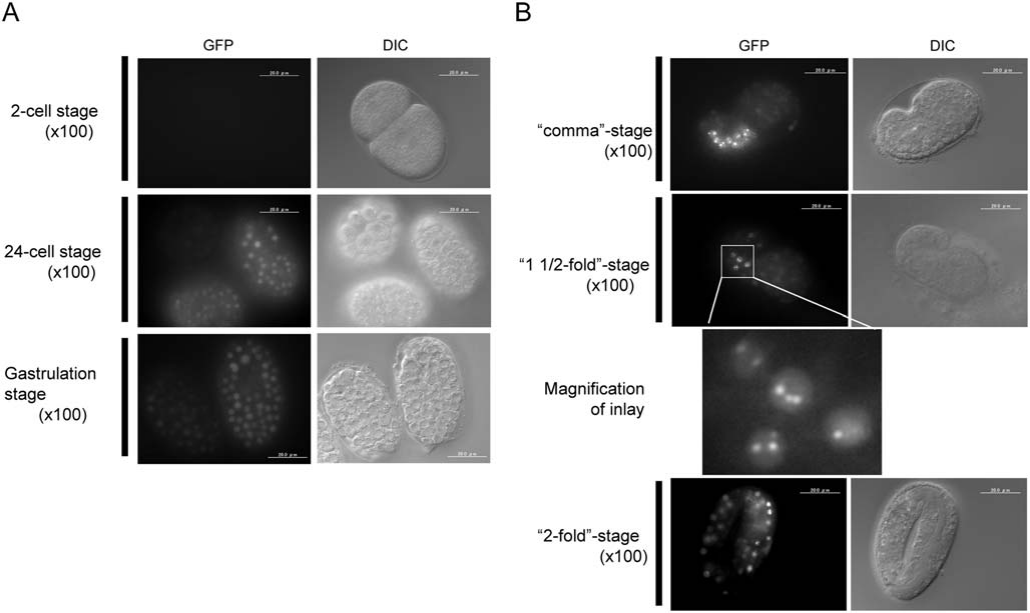
Pqbp-1.1-Venus fusion protein expression during early embryonic stages. A) Expression was detected after 24-cell stage. Pqbp-1.1-Venus was expressed in all cells until gastrulation stage. The fluorescence negative area corresponds to gastrulation lumen. B) From comma stage, expression of pqbp-1.1-Venus focused to intestine.

**Figure 3 pone-0004104-g003:**
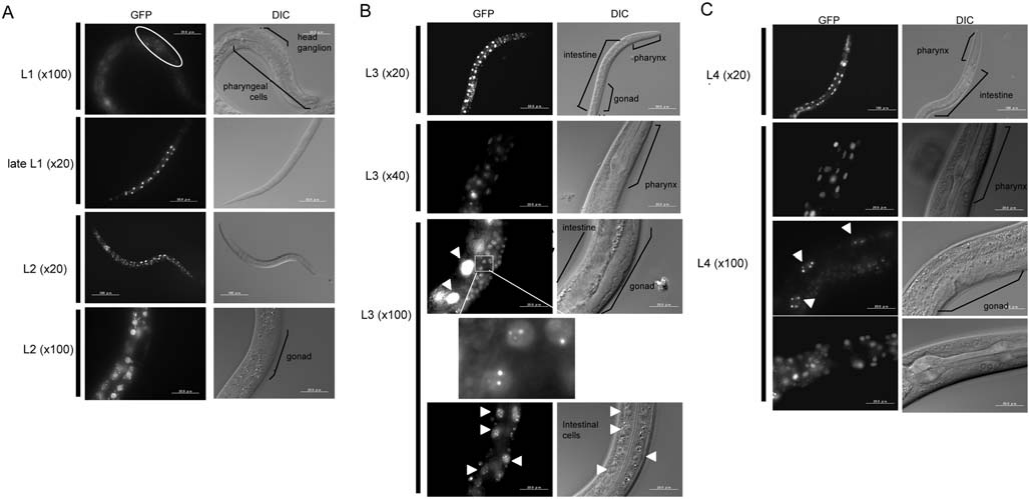
Pqbp-1.1-Venus fusion protein expression during larva stages. A) At L1 stage, pqbp-1.1-Venus fusion protein is expressed in progenitor neurons in head ganglion. Expression in intestinal cells increases from L1 to late L1 stgae. At L2 stage, pharyngeal and intestinal cells show strong signals. Gonad does not express the fusion protein. B) At L3 stage, pharyngeal and intestinal cells keep a high expression level of pqbp-1.1. Especially, intestinal cells the highest expression level during the development. Somatic gonads also show high signals, and a higher magnification reveals nuclear dots of fusion protein, which is similar to the intranuclear localization of mammalian PQBP1 [11]. Arrowheads indicate intestinal cells. C) At L4 stage, a small number of pharyngeal and intestinal cells show strong signals of pqbp-1.1-Venus. The nuclear dots of the fusion protein are observed in intestinal cells (arrowheads) similarly to L3 stage. Somatic gonads also show weak signals.

By injecting the reporter plasmid, we obtained F2 transgenic worms as described in *Materials and Methods*. The fluorescence signals of the pqbp-1.1-Venus fusion protein indicated that pqbp-1.1 was expressed from 24-cells stage to Larva stage 4 (L4) (Fig. 2, 3). From 24 cells- to gastrulation-stage, pqbp-1.1-Venus was expressed ubiquitously (Fig. 2). At L1 stage, neural cells in the head ganglion express the fusion protein (Fig. 3A). From late L1 stage, intestinal cells expressed pqbp-1.1-Venus strongly and the high expression continued to L4 stage. The peak of expression was observed at L3 stage. Pharyngeal cells show an almost similar pattern of expression during development, while the peak seems to be at L4 stage (Fig. 3C). Higher magnification of the signals revealed that the fusion protein formed speckle-like intranuclear dots (or nuclear bodies) just like mammalian PQBP1 [2], [11] (Fig. 3B, 3C).

**Figure 4:**
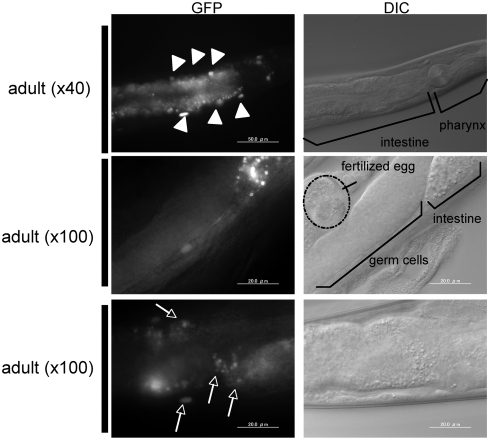
Pqbp-1.1-Venus fusion protein expression in adult nematode. Expression in the pharynx decreases. Intestinal cells keep a high expression level of pqbp-1.1-Venus in adult worms. Arrowheads indicate intestinal cells. Somatic gonads show no expression of pqbp-1.1-Venus. In the tail region, fluorescence was detected in several neurons (arrows).

After becoming adult worm, pqbp-1.1-Venus expression reduced rapidly in pharynx, while the expression continued in intestinal cells (Fig. 4).

### PQBP1 expression in somatic gonads and head ganglions

During the development, somatic gonad cells expressed pqbp-1.1-Venus only from L3 to L4 stage (Fig. 3B, C), and adult gonads or fertilized eggs did not express pqbp-1.1-Venus (Fig. 4).

*Pqbp*-1.1 expression was also observed in head ganglion cells at L1 stage. The expression in such neural precursor cells is consistent with the developmental expression pattern of PQBP1 in mice [14]. Some neurons in the tail show weak expression of pqbp-1.1-Venus in adult worms (Fig. 4). However, after L1 stage, expression in neurons was relatively weak in comparison to that in intestinal cells or somatic gonad, and further analysis is needed to clarify the detail.

### Depletion of PQBP1 homologue leads to reduction of lipid storage

**Figure 5:**
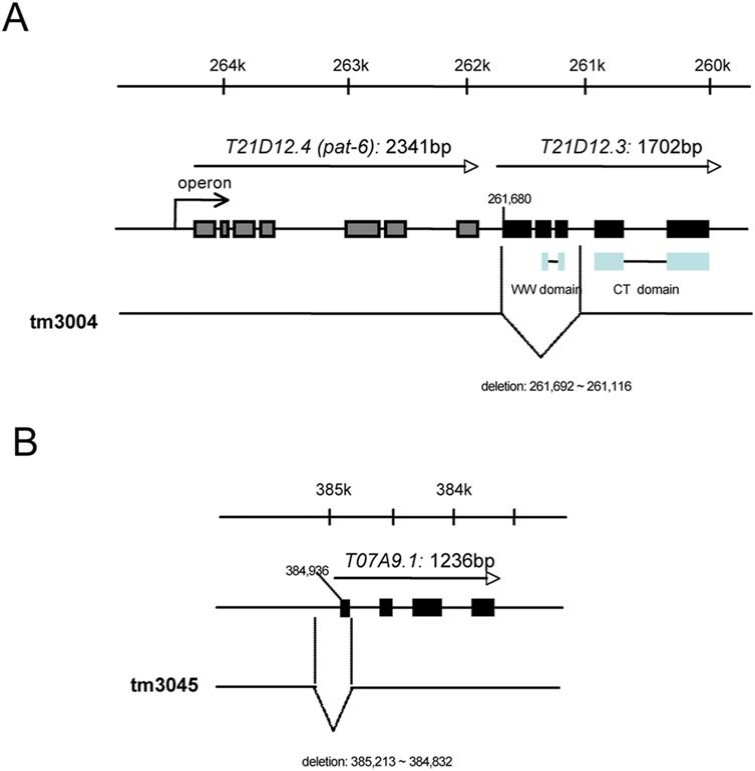
Generation of *T21D12.3* -specific and *T07A9.1* -specific mutant nematodes. A) *T21D12.3* is the strictly conserved homologue of mammalian *PQBP1* possessing WW domain and C-terminal domain. The genome maps show deleted regions in the genome of *T21D12.3* and *T07A9.1* mutants. B) *T07A9.1* possesses a homology only to C-terminal domain of PQBP1 and does not possess WW domain. The genome maps show deleted regions in the genome of *T07A9.1* mutants.

Screening of the *T21D12.3* mutants was performed as described previously [15]. Consequently, a mutant (*tm3004*) was obtained from the screening, which lacks a part of the *T21D12.3* genome including the first methionine sequence (Fig. 5A). Because the expression of *T21D12.3* was rather confined to intestinal cells (Fig. 3, 4) and because intestinal cells play critical roles in nutritional state of nematode [16], we investigated intestinal lipid storage of these mutants in this study.

**Figure 6:**
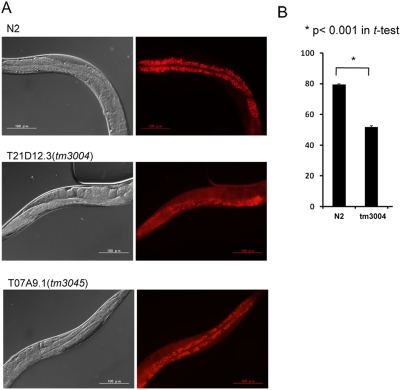
Morphological analysis of lipid storage in intestinal cells. A) Worms were stained by culturing from the L1 stage to adult stage on a Nile Red-containing plate. Control worms (N2) show strong stains in intestinal cells. In the *T21D12.3* mutants, Nile Red stains intestinal cells weakly and the stain signals in body cavity outside of eggs seem higher than that of N2. Nile Red stains in the *T07A9.1* mutants were not changed so remarkable. B) Nile Red staining signals in intestinal cells were analyzed quantitatively by collecting signals from 6 worms (25 µm^2^). Asterisks indicate p<0.001 in Student's t-test. Quantitative analysis showed a significant difference between N2 and *T21D12.3* mutants but not between N2 and *T07A9.1* mutants.

To take advantage of lucent body of C. elegans, lipid storage was investigated by culturing on Nile Red-including dishes. It is known that the *in vivo* staining with Nile Red does not affect growth, feeding or lifespan of C. elegans [17]. The Nile Red signals in intestinal cells were clearly lower in homozygous *tm3004* than in wild type N2 (Fig. 6A, B) although the number of intestinal cells did not change (Fig. 6A).

**Figure 7:**
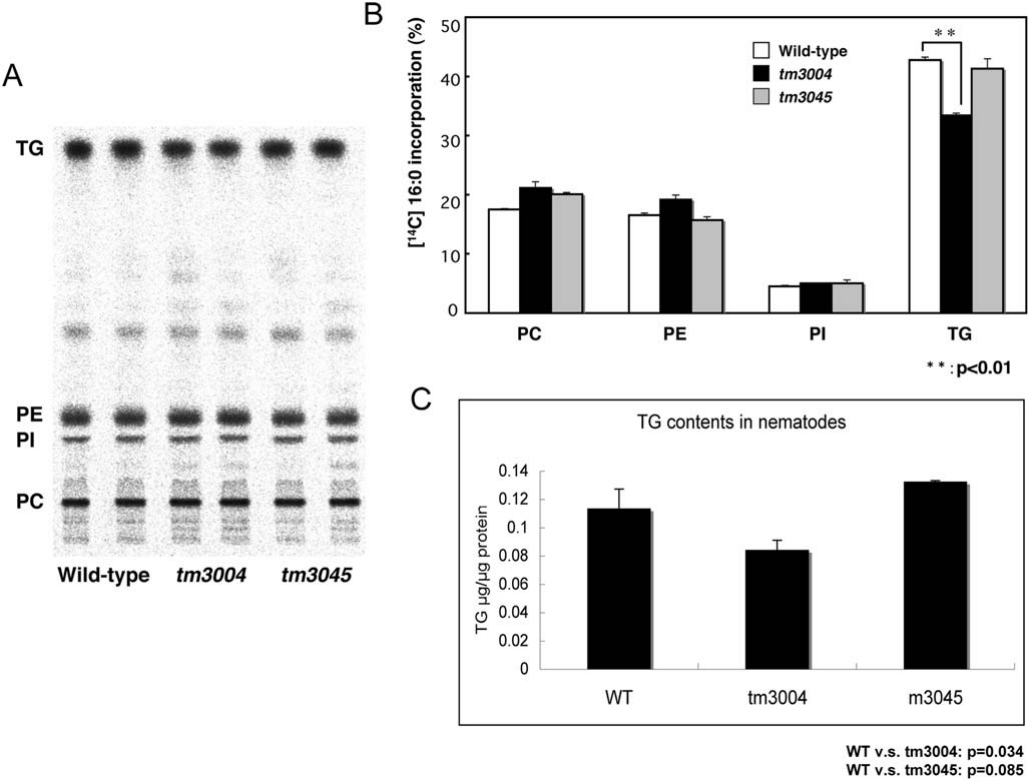
Abnormal lipid metabolism in the PQBP1 homologue mutant. A) Uptake of exogenous radio-labeled fatty acid (16∶0) into various lipids in normal (N2), homozygous tm3004 mutant, and homozygous tm3045 mutant nematodes. TLC was exposed to for 10 hours. B) Quantitative analysis of the results in Fig. 7A. C) Triglyceride contents in nematodes.

To confirm these semi-quantitative results regarding lipid contents, we investigated the uptake of exogenous radio-labeled fatty acid (16∶0) into various types of lipids (Fig. 7A, B) and the total content of triglyceride (Fig. 7C) in the homozygous *tm3004* mutants. The *tm3004* mutant shows a decrease in uptake of fatty acid specifically into triglyceride, while the uptake into other phospholipids was not affected. The total content of triglyceride was also decreased (Fig. 7C). These results supported that the *T21D12.3* mutation caused reduction of triglyceride, and suggest that the reduction was induced by either impaired synthesis or enhanced degradation of triglyceride.

### Depletion of PQBP1 paralogue does not lead to reduction of lipid storage

In addition to *T21D12.3*, the genome of C. elegans encodes another paralogue, *T07A9.1* (pqbp-1.2), possessing a low homology to PQBP1 but lacking WWD. Thus, we similarly screened and obtained a mutant of *T07A9.1* (*tm3045*) lacking a part of the gene (Fig. 5B). However, the mutant nematodes did not show reduced signals in Oil Red staining (Fig. 6A), abnormal uptake of radio-labeled fatty acid (16∶0) into triglyceride (Fig. 7A, B) or reduction of triglyceride contents (Fig. 7C). Therefore, pqbp-1.1 but not pqbp-1.2 seems to be a proper orthologue of PQBP1 at least from the aspects of molecular structure and lipid metabolism.

### PQBP1 is involved in lipid metabolism of mammalian white adipocytes

We finally tested whether findings in C. elegans were applicable to mammalian adipocytes. Reviewing our previous in situ hybridization data confirmed expression of PQBP1 in fat tissues [14]. Primary white adipocytes prepared from newborn mice were transfected during growth phase with a plasmid PQBP1-shRNA-ZsGreen, which express both mouse PQBP1-shRNA and ZsGreen. We also used non-silencing shRNA-ZsGreen transfected and non-transfected cells as controls.

**Figure 8:**
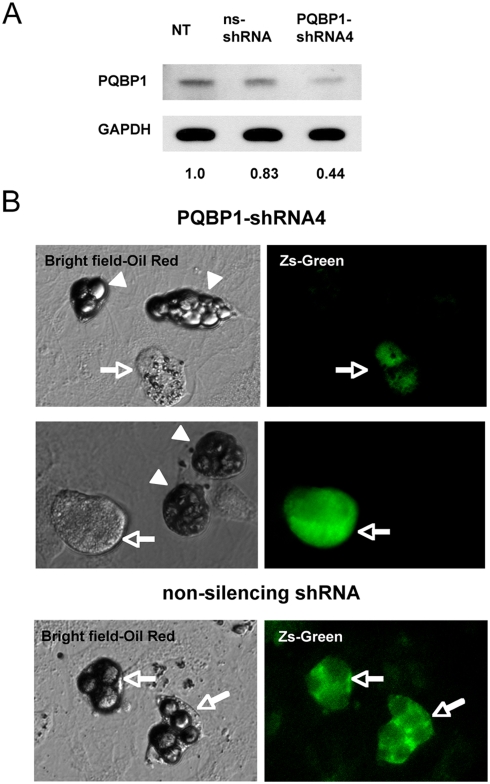
ShRNA-mediated suppression of PQBP1 inhibits lipid storage in white adipocytes. A) Western blot shows that PQBP1-shRNA-IRES-ZsGreen plasmid suppresses PQBP1 protein in primary mouse white adipocytes. Non-silencing shRNA (ns-ShRNA) was used as a control. The band intensities are corrected with GAPDH and standardized with non-transfected cells (NT). B) Primary white adipocytes were stained by Oil Red after transfection with PQBP1-shRNA-ZsGreen or non-silencing shRNA-ZsGreen plasmid. Non-transfected cells (arrowheads) possess large lipid droplets while PQBP1-shRNA-transfected cells (green) show small granules (arrows). Non-silencing shRNA-transfection did not affect the size of lipid granules (arrows in lower panels).

PQBP1-shRNA actually reduced PQBP1 protein in western blots with anti-PQBP1 antibodies (Fig. 8A). We observed lipid granules were decreased in PQBP1-shRNA expressing fluorescence-positive cells (arrows) in comparison to fluorescence-negative cells (arrowheads) in the same plate. Both the sizes and Oil Red staining intensities of lipid droplets were significantly reduced (Fig. 8B). Non-silencing shRNA / ZsGreen-expressing cells also did not show any significant change (Fig. 8B). These results collectively support that mammalian PQBP1 also decrease the lipid content.

## Discussion

### Nematode PQBP1 homologue is involved in lipid metabolism

The method used in this study, which detects a marker fusion protein (PQBP1-Venus) expression under the control of the endogenous enhancer/promoter, definitely shows the endogenous expression pattern of PQBP1 in C. elegans. The expression pattern of nematode PQBP1 was actually unexpected, since we had expected to observe a neuron-dominant expression pattern. However it is not rare that a molecule is differentially used in different organisms even if it is highly conserved beyond species. Moreover, PQBP1 is highly conserved in Arabidopsis although the plant does not have the brain.

Our results clearly showed that nematode pqbp-1.1 is expressed in cells for lipid-storage (Fig. 1–4) and that pqbp-1.1 depletion links to abnormal lipid metabolism in nematode (Fig. 5, 6). Mammals possess fat cells to store lipid in the body, while nematode keep lipid in intestinal cells. However, although the cell types for lipid storage are different, compositions of lipid (such as saturated or unsaturated fatty acid, triglyceride) and metabolic pathways are fundamentally conserved beyond species. In addition, the knock down experiment with shRNA showed reduction of lipid storage in mammalian white adipocyte (Fig. 7). Therefore, the mutant nematodes generated in this study could be a hypothetical model for the lean body symptom of human patients linked to PQBP1 mutations. This model will be available for further analysis of the detailed molecular mechanisms underlying the lean body, and such studies might develop PQBP1 to a novel target molecule in human lipid metabolism.

### Lipid metabolism in PQBP1 and polyQ pathologies

PQBP1 is known to interact with mutant Htt [18]. Meanwhile, mutant huntingtin (Htt), the causative gene product of Huntington's disease (HD), is known to downregulate peroxisome proliferator-activated receptor gamma coactivator 1 alpha (PGC-1α) [19]–[21], a co-activator of peroxisome proliferator-activated receptor (PPAR-γ) that is essential for transcription of a number of genes involved in glucose metabolism and β-oxidation of fatty acid [for review see 22]. Interestingly, in addition to binding to the promoter, PGC-1α participates in RNA elongation and splicing through binding to factors anchored onto C-terminal domain of RNA polymerase II [22]. The binding property of PGC-1α to RNA polymerase II and splicing proteins is homologous to that of PQBP1 [2], [4], suggesting a possible relationship between PQBP1 and PGC-1α. Therefore, it might be worth analyzing whether PQBP1 is involved in the same transcription protein complex with PGC-1α and how PQBP1 affects lipid metabolism mediated by PGC-1α in normal physiology and in HD pathology.

A hypothesis to account for the change of lipid granules would be that PQBP1 regulates expression of Fsp27/CIDE-C, which locates at lipid droplets and influences their size [23], like PPAR-γ regulates Fsp27 [24]. CIDE-A, another lipid droplet-associated protein controlling triglyceride deposition in white adipocytes [25] might be another target of transcriptional control by PQBP1. Though we have obtained preliminary data to support the hypothesis (data not shown), these issues would be the next project regarding the lipid metabolism function of PQBP1.

### PQBP1 expression in neurons of C. elegans

In this study, we focused on the lipid metabolism, and have not analyzed neuronal functions of the nematode homologue of PQBP1. However, in mammals, PQBP1 definitely functions in neurons and/or progenitor cells, and the neuronal functions are essential for understanding MR symptoms found in PQBP1-mutated patients. Here, we found that pqbp-1.1 was expressed in head ganglion cells at L1 stage (Fig. 3A), which may correspond to neural progenitor cells in mammals. Although our preliminary observation showed no remarkable decline of locomotive activity, further analysis is needed for definite evaluation and it is essential for understanding *T21D12.3* function in learning and memory functions in C. elegans. Meanwhile, analysis of PQBP1 functions in neurons are now undergoing in our laboratory with Drosophila mutants and mammalian neural stem cells. Drosophila mutants show a unique type of learning impairment (unpublished observation), which is different from the long-term memory disturbance observed in transgenic fly overexpressing human PQBP1 [26]. These experiments using various animal models will promote understanding of the whole scheme of PQBP1 diseases and might lead to certain insights into basic biology in the future.

Human patients with mutations of the PQBP1 gene show mental retardation and microcephaly as major symptoms. However, PQBP1 is expressed widely in multiple tissues of mammals. Therefore, if different animal models have different advantages for analyzing multiple molecular functions of a gene, we should better take the merit. We should employ appropriate animal models for appropriate functional analyses. In this study, we took advantage of the nematode model for analyzing the phenotype in lipid metabolism. We have already constructed conditional knock out mice and RNAi-mediated knock down mice as well as fly models for analyzing neuronal functions of PQBP1, which will be reported elsewhere.

The “lean body” is not a tiny symptom because the research on this symptom might link to cell metabolism and the related disorders. Although the phenotype we observed in this study might be a downstream consequence of essential molecular functions of PQBP1, still this work shows a novel finding to suggest a possible involvement of PQBP1 in the lipid metabolism.

## Materials and Methods

### Plasmid construction for transgenic C. elegans expressing venus under the control of *T21D12.3 enhancer/promoter*

An about 6.7 kb DNA fragment containing an operon of T21D12.2 (*pat-6*) and T21D12.3 gene was amplified from N2 genome DNA with primers 5′-GCCTATCAATATCAGGCACA and 5′-CTTCGTCCCACCTACATAAC (Fig. 1) with KOD plus (Toyobo). One Adenine wad added to the end of the amplified DNA fragment during reaction. The genome fragment was subcloned into pGEMT-T Easy vector (Promega) and the resultant plasmid (pGEMT-T-T21D12.3) was digested with Pml I (BioLabs) which is unique in the exon 4 of the T21D12.3 gene. Venus gene cDNA was amplified from pFX-Venus vector [13] with primers 5′-ATGATGATAAGCAATCACGTGGAATGGTGAGCAAGGGCG and 5′-TTTCAGCATTATTGCCACGTGTCTTGTACAGCTCGTCCATGC, then the fragment was subcloned into the Pml I site of pGEMT-T-T21D12.3 with recombinase in-Fusion kit (Clontech). The Venus Open Reading Frame was adjusted to that of T21D12.3 by inserting “GA” to 5′ and “T” to 3′ end of Venus cDNA, giving rise to the plasmid was named pGEMT-T-T21D12.3-Venus. The junctions between T21D12.3 and Venus were confirmed by sequencing with primers AGAAATTTCCACAACAAGAA and TCTGGACCCTGTGACGTCAT.

### Generation of transgenic C. elegans

pGEMT-T-T21D12.3-Venus (20 µg/ml) and pFX-RXT-myo-2 (20 µg/ml), a marker of pharyngeal muscle to distinguish transgenic worms, were mixed and injected into the germ line of adult worm, according to the method by Mello et al. [27]. After 3 days, fluorescence-positive F1 worms were picked up, and transferred to a new dish. After additional culture for 3–4 days, fluorescence-positive F2 worms were selected and named *tmEx1642*.

### Screening of mutant C. elegans of *T21D12.3* and *T07A9.1*

The deletion mutations used in this work were isolated as previously described [15]. Primers used in nested PCR screening for detecting *T21D12.3 (tm3004)* are as follows; first round: 5′-CCGGCGGCGATACCCATTAA-3′, 5′-CCTAATCAAACGACACCGTC-3′, second round: 5′-GCGATACCCATTAAGTACGA-3′, 5′-GTCTGGCTCAACAACTATGG-3′. Primers for screening *T07A9.1 (tm3045)* are as follows; first round: 5′- TCGCAAGTCCCTCATCATAG-3′, 5′- CGAAATAACTGTGTGCGCCT-3′, second round: 5′- ACAGTGATGACGTCGTCTAG-3′, 5′- CTATTGCGATTGAGATGCTC-3′. Sequence analysis of the deleted regions was performed as previously described. These deletion alleles were outcrossed at least twice.

After screening of the mutants, we obtained a line for *TD21D12.3* (*tm3004*) and another line for *T07A9.1* (*tm3045*). Deleted regions in the genome of these mutants were determined by sequencing of the PCR fragments from their genomes. The primers for *TD21D12.3* were 5′-CCGGCGGCGATACCCATTAA and 5′-CCTAATCAAACGACACCGTC. The primers for *T07A9.1* were 5′-CTGTACATATCTCTCGGGCA and 5′-AGGTCGTGCGTTGAATGAGA.

### Nile Red staining of intestinal cells and morphological observation

Nile Red (5H-benzo[a] phenoxazine-5-one, 9-doethylamino) was solved in acetone at 500 µg/ml and diluted to 2.5 µg/ml with phosphate-buffered saline. 3 ml of the solution was layered on a 35 mm NGM plate and dried at room temperature. Starved worms were cultured for 3 days before observation. Microscopic images were obtained by Olympus BX-50 and Olympus SenSys CCD camera. Fluorescence of Nile Red was observed with a Rhodamine filter (580 nm). Nile Red staining images were obtained at the same magnification (20×) and same exposure time (260 msec). Signal intensities in the images were quantified using Aquacosmos (HAMAMATSU). The arbitrary signals of intestinal cells were subtracted by those of body cavity to standardize the intensities. More than 100 cells were quantified in each type of nematode.

### Lipid uptake and content analyses with nematodes

Incorporation of exogenous [1-^14^C] stearic acid into *C. elegans* was analyzed as described previously [28]. For triglyceride analysis, lipids of synchronized young adult worms were extracted by the method of Bligh and Dyer [29], and the triglyceride contents were measured using enzymatic kit (Triglyceride E-test, Wako Pure Chemical Ltd.) according to the manufacturer's protocol.

### PQBP1-shRNA plasmid construction

The shRNA expression plasmids against mouse PQBP1 was derived from the RNAi-Ready pSIREN-RetroQ-ZsGreen (Clontech) shRNA expression vector. The double-stranded DNA oligonucleotides encoding shRNA against PQBP1 were composed of a sense strand, a 9-nucleotide hairpin loop, the complementary anti-sense strand, a poly(A) termination signal and an Xho I site. The sequence was derived from mouse PQBP1 specific siRNAs (Qiagen):GAGGAAGAGATTATTGCTGAA (SI01387120). The synthesized double strand oligonuclotides containing the sense and anti-sense siRNA sequences (5′-GGATCCGGAGGAAGAGATTATTGCTGAATTCAAGAGATTCAGCAATAATCTCTTCCTCCTTTTTTCTCGAGGAATTC-3′) were inserted into the BamH I and EcoR I sites of the shRNA expression vectors at the 5′- and 3′-ends, respectively. A control non-silencing shRNA (GGATCCGTGCGTTGCTAGTACCAACTTCAAGAGATTTTTTACGGCGTGAATTC) was synthesized and inserted into the shRNA expression vector.

### Primary culture of white adipocytes and shRNA transfection

White adipocytic precursor cells were obtained from Primary Cell Co., Ltd (Hokkaido, Japan). The cells were grown in growth medium (Dulbecco's Modified Eagle Medium, 10% foetal bovine serum, 10unit/ml penicillin, 10 µg/ml streptomycin, 17 µM pantothenic acid, 33 µM (+)-biotin, 100 µM ascorbic acid, 1 µM octanoic acid, and 50 nM Triiodothyronine) at 37°C and 5% CO_2_ for 24 hours. The cells were transferred to 10 cm dish pre-coated with collagen at a density of 2×10^4^cells/cm^2^ and incubated for 12 hours. Thereafter, the cells were transfected with PQBP1 shRNA-ZsGreen plasmid using Effecten (Qiagen) and incubated for 12 hours. Transfection with a non-sense shRNA plasmid and the mock treatment were performed in parallel. Subsequently, the medium was changed to Dulbecco's Modified Eagle Medium containing 10% fetal bovine serum, 10unit/ml penicillin, 10 µg/ml streptomycin, 17 µM pantothenic acid, 33 µM (+)-biotin, 100 µM ascorbic acid, 1 µM octanoic acid, 50 nM Triiodothyronine, 10 µg/ml Insulin, and 2.5 µM Dexamethasone for 48 hours to differentiate the cells to white adipocyte. Finally, the cells were kept for 7 days in maintenance medium (Dulbecco's Modified Eagle Medium, 10% fetal bovine serum, 10unit/ml penicillin, 10 µg/ml streptomycin, 17 µM pantothenic acid, 33 µM (+)-biotin, 100 µM ascorbic acid, 1 µM octanoic acid, 50 nM Triiodothyronine, and 10 µg/ml Insulin) before image acquisition.

### Oil Red staining of white adipocytes

White adipocytes were fixed two times with 4% paraformaldehyde in phosphate buffer, pH 7.4, for 15 min and 10 min, washed with PBS, and treated with 60% isopropanol for 1 min. Next, the cells were incubated with Oil Red for 10 min at 37°C. After Oil Red staining, cells were treated with 60% isopropanol for 2 min and washed three times with PBS.

### Western blot analysis of white adipocytes

Western blots were performed with anti-PQBP1 rabbit antibody against human 1–265 a.a. that cross-reacts with mouse PQBP1 (sc-32910, Santa Cruz). Western blot with the antibody showed a single band confirming the specificity (data not shown).
